# Contrast-enhanced ultrasound of Crohn’s disease in children and young adults: quantitative metric correlations and MRI disease severity associations

**DOI:** 10.1007/s00247-025-06203-8

**Published:** 2025-03-13

**Authors:** Jonathan R. Dillman, Adam F. Prasanphanich, Katherine N. Epstein, Alexander J. Towbin, Andrew T. Trout

**Affiliations:** 1 Department of Radiology, Cincinnati Children’s Hospital Medical Center, 3333 Burnet Avenue, Cincinnati, OH 45244 USA; 2 University of Cincinnati, Cincinnati, USA

**Keywords:** Children, Contrast-enhanced ultrasound, Crohn’s disease, Inflammation, Inflammatory bowel disease, Magnetic resonance imaging, Quantitative, Ultrasound

## Abstract

**Background:**

There is a paucity of data comparing contrast-enhanced ultrasound (CEUS) to MR enterography in children and young adults with Crohn’s disease.

**Objective:**

To measure correlations across CEUS quantitative metrics in children and young adults with Crohn’s disease, and to evaluate if these metrics are associated with MRI features of disease activity.

**Materials and methods:**

Patients <21 years old with Crohn’s disease affecting the terminal ileum who underwent clinically-indicated MR enterography were recruited between 2021 and 2024. Research CEUS of the terminal ileum was performed using sulfur hexafluoride lipid-type A microspheres, and images were analyzed using VueBox (Bracco Group). MRI exams were independently reviewed by three radiologists to document features of disease activity. Pearson’s correlation (*r*) was used to measure associations across nine CEUS quantitative metrics and between CEUS metrics and mean or consensus MRI features.

**Results:**

Twenty-five participants, 13 (52%) male, with a mean age of 16.5 years (range, 13-20 years) were included. The mean terminal ileal maximum bowel wall thickness at MRI was 7.5 mm±1.8 mm. The mean sMaRIA score was 3.4±0.8, consistent with severely active disease. CEUS quantitative measurements were highly variable across participants. The mean rise time was 7.0±2.7 s, while the mean peak enhancement was 3,282±3,754 a.u. Twelve of 36 (36%) CEUS quantitative metric bivariate comparisons were highly collinear with *r*>0.8 (*P*<0.0001). There were significant positive correlations between CEUS rise time and MRI maximum bowel wall thickness (*r*=0.40; *P*=0.046), visual analog scale assessment of overall inflammation (*r*=0.43; *P*=0.032), and postcontrast enhancement ratio (*r*=0.47; *P*=0.018); there were no other significant correlations between CEUS metrics and MRI measures of inflammation.

**Conclusion:**

CEUS quantitative measurements are highly variable across patients with Crohn’s disease, with multiple metrics being highly correlated with one another. CEUS rise time correlates with MRI features of disease activity.

**Graphical Abstract:**

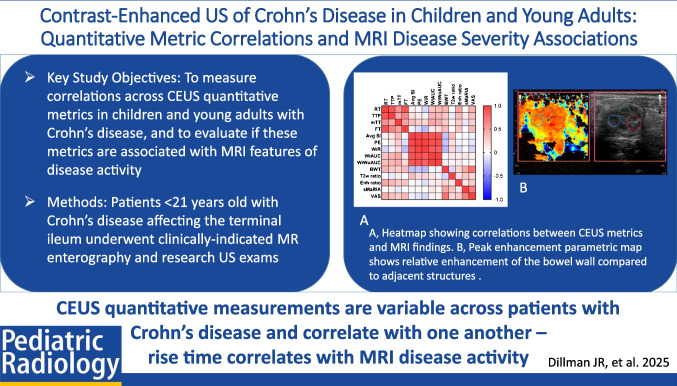

## Introduction

Imaging plays a central evidence-based role in the diagnosis and monitoring of Crohn’s disease in both children and adults [1, 2]. It is essential for determining the extent and severity of active intestinal inflammation, as well as for detecting and characterizing associated complications. The most used imaging modalities for evaluating CD are CT enterography (CTE) and MR enterography (MRE); however, the use of ultrasound is on the rise due to its potential as a more accessible, lower-cost diagnostic tool. Much of this increased use in North America has occurred in the pediatric and adult gastroenterology clinic settings [3]. Ultrasound can be performed more frequently than CTE and MRE, making it an appealing option for ongoing monitoring of patients with CD, potentially enabling real-time bedside clinical decision-making [4]. This closer observation of intestinal disease activity may aid in the achievement of transmural imaging and histologic healing, increasingly recognized likely important treatment targets in CD management [5].

A variety of ultrasound methods can be employed to detect intestinal inflammation, including gray-scale (B-mode) anatomic evaluation, Doppler blood flow analysis, and motility assessment, all of which can be conducted without the need for intravenous contrast material [6, 7]. Ultrasound findings have also been shown to change over time in response to medical therapy [8, 9]. Recently, ultrasound microbubble contrast agents, such as sulfur hexafluoride lipid-type A microspheres, have become increasingly available, facilitating the use of contrast-enhanced ultrasound (CEUS) techniques [10]. However, there remain few studies exploring the application of CEUS in patients with CD, particularly children [11–16]. Pediatric studies are notably small to date, and some use only qualitative assessments (despite CEUS being a potentially more objective, quantitative diagnostic tool) [11, 17]. Research is needed to understand how various CEUS quantitative metrics correlate with one another; demonstrating collinearity could potentially streamline the number of metrics used in clinical practice. Additionally, it is important to investigate how these CEUS quantitative metrics relate to accepted MRI features of disease activity to enhance clinical understanding and adoption.

The purpose of this study was twofold: first, to measure the correlations across various CEUS quantitative metrics in children and young adults with CD; and second, to evaluate whether these metrics are associated with MRI features of disease activity.

## Methods

The prospective study received institutional review board approval and was conducted in compliance with HIPAA regulations. Oral and written informed consent were obtained from all participants 8 years of age and older, while informed assent was secured for those under 18 years of age with consent provided by their parent or legal guardian.

### Study sample

Children and young adults up to 21 years of age with a confirmed diagnosis of CD affecting the terminal ileum based on prior endoscopy and biopsy, who had recently undergone a clinical MRI enterography (MRE) exam, were recruited for the study between April 2021 and June 2024. Individuals were excluded if they were known or suspected of being pregnant, had previously undergone resection of the terminal ileum, had a known allergy to an ultrasound contrast agent, or were unable to tolerate abdominal ultrasound scanning due to pain or discomfort. All individuals were on some form of standard of care medical treatment at the time of study participation; length of disease duration and the degree of terminal ileal abnormality on the clinical MRE were not considerations for study enrollment.

Demographics, height and weight, and laboratory inflammatory markers (e.g., C-reactive protein, erythrocyte sedimentation rate) were documented on the day of the research CEUS examination. The Harvey-Bradshaw Index (HBI), a clinical tool used to assess disease activity in patients with CD, was also measured at the time of the research visit, where remission <5, mild disease 5–7, moderate disease 8–16, and severe disease >16 [18]. The dates of diagnosis and CD-related medical therapy were documented using electronic health records.

### CEUS performance

Research dynamic CEUS exams of the terminal ileum were conducted using an Aplio i800 ultrasound system (Canon Medical Systems) and 14L5 transducer by the same operators, including a single fellowship trained pediatric radiologist with expertise in bowel imaging, a single ultrasound technologist with expertise in intestinal ultrasound, and a small group of research nurses that administered the IV contrast material (under the supervision of the study radiologist). After placing a peripheral IV cannula in the upper extremity (20–22 gauge), sulfur hexafluoride lipid-type A microspheres (Lumason; Bracco Group) were bolus injected by hand at approximately 1 ml/s, followed by a 10 ml normal (0.9%) saline flush, also injected at 1 ml/s. The contrast material was administered according to U.S. FDA-approved dosing guidelines, based on weight (0.03 ml/kg), with a maximum dose of 2.4 ml. Images were acquired using the CEUS mode on the ultrasound system, with low mechanical indices ranging from 0.06 to 0.13. Cine imaging was performed continuously for 120 s, with the recording of images starting immediately upon the completion of the intravenous saline flush. A second bolus of IV contrast material could be administered if warranted based on our approved study protocol (e.g., incorrect or suboptimal ultrasound system parameter(s) selection, catheter or tubing malfunction affecting the bolus injection of contrast material). Imaging was performed in the optimal plane (short vs. long-axis) to maximize bowel wall visualization.

### CEUS image analysis

**Fig. 1:**
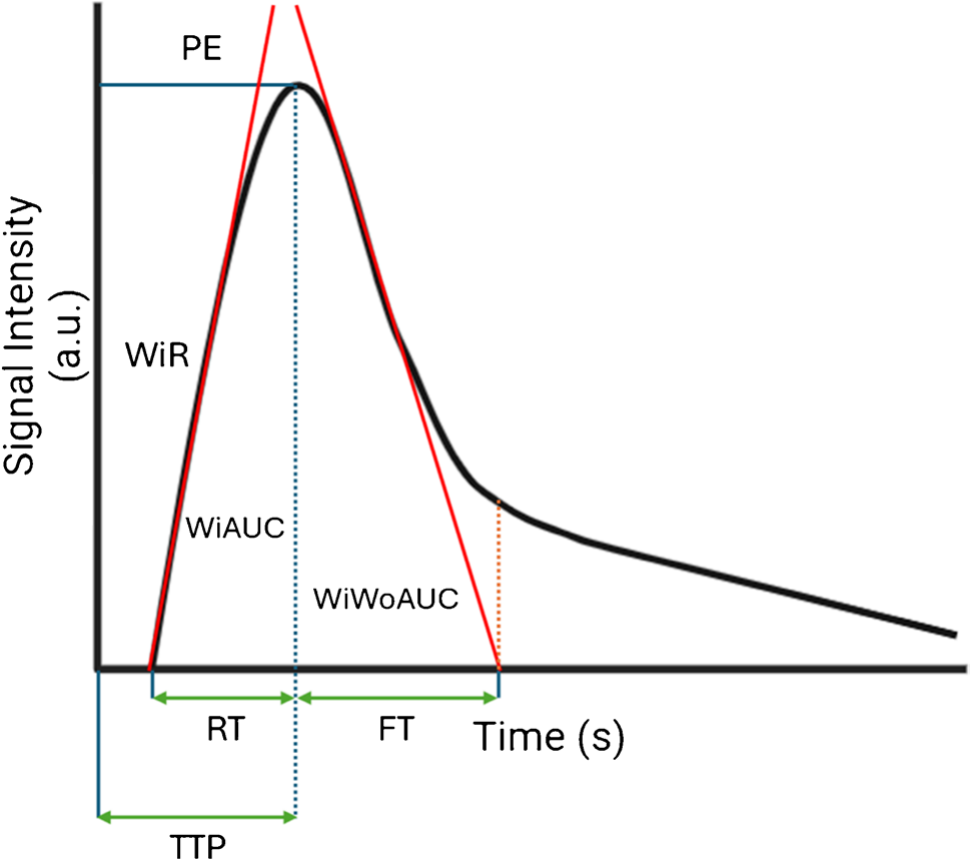
Contrast-enhanced ultrasound perfusion time versus signal intensity curve. FT, fall time; PE, peak enhancement; RT, rise time; TTP, time to peak; WiR, wash-in rate; WiAUC, wash-in area under the curve (area under the curve from the start of contrast material wash-in to the time of bowel wall peak enhancement [*dotted blue line*]); WiWoAUC, combined wash-in and wash-out area under the curve (area under the curve from the start of contrast material wash-in to the end of fall time [*dotted orange line*])

**Fig. 2:**
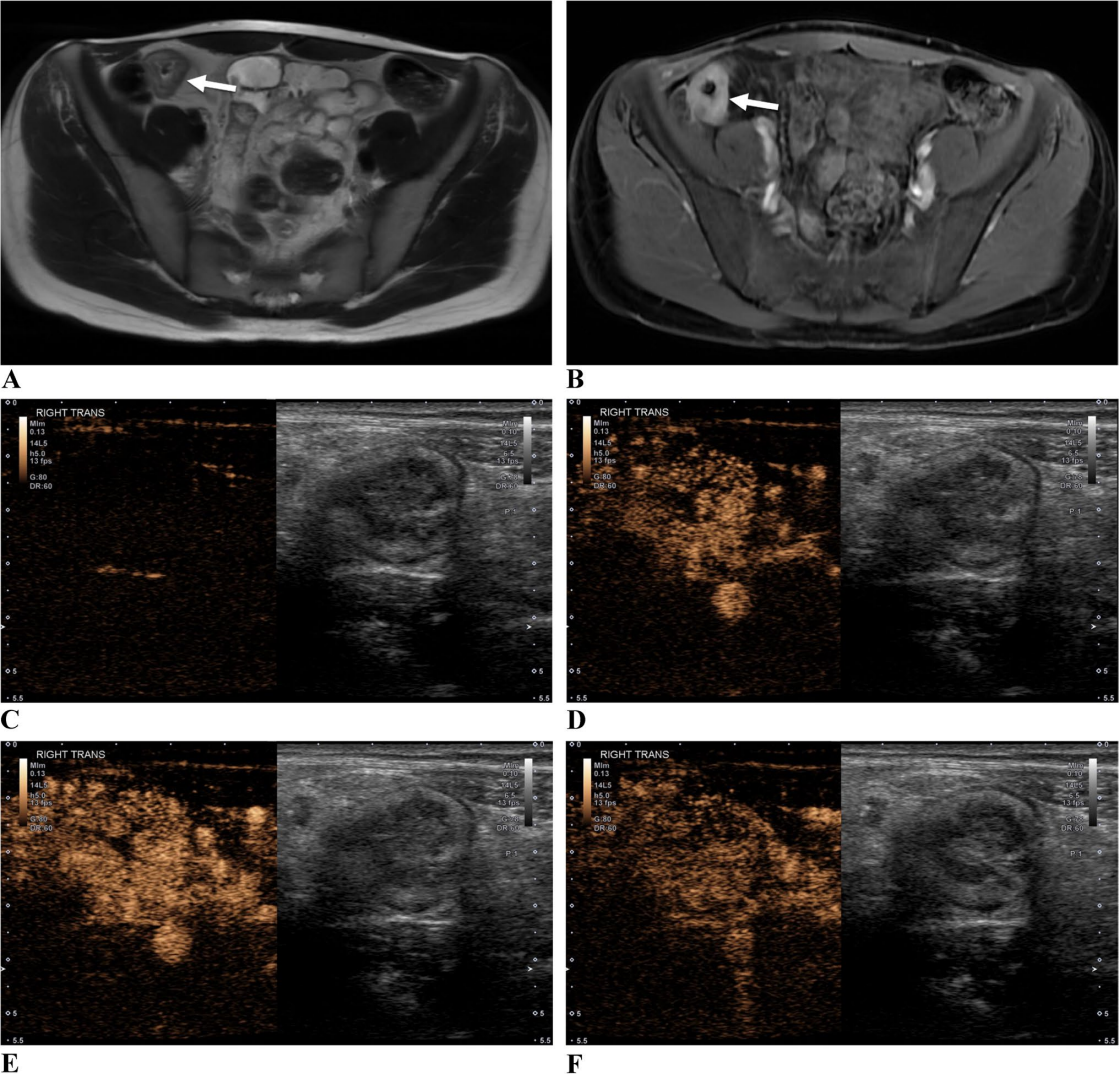

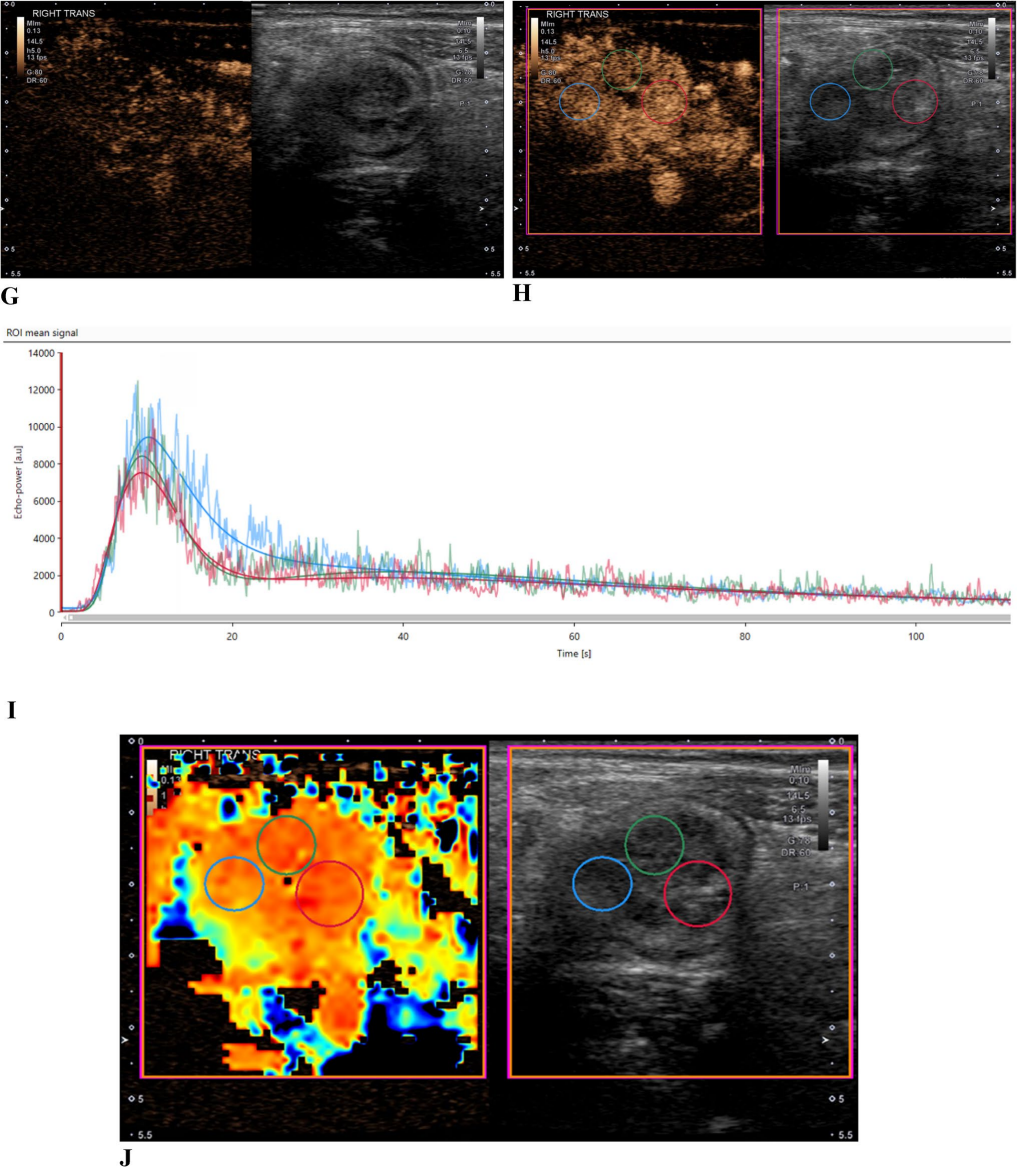
A 19-year-old with ileal Crohn’s disease. **a** Axial single-shot fast spin-echo MR image shows terminal ileal wall thickening and intramural edema (arrow). **b** Axial postcontrast T1-weighted image shows marked hyperenhancement (arrow). **c**-**g** Side-by-side contrast-specific and gray-scale contrast-enhanced ultrasound images at 0 s, 15 s, 25 s, 60 s, and 120 s show wash-in and wash-out of contrast material in the bowel wall over time. **h** Ultrasound image at peak enhancement shows three round regions of interest in the bowel wall. **i** Time vs. signal intensity curves from the three regions of interest show bowel wall wash-in and wash-out of microbubble contrast agent. **j** Contrast-enhanced ultrasound peak enhancement parametric map shows relative enhancement of the bowel wall compared to adjacent structures

**Fig. 3:**
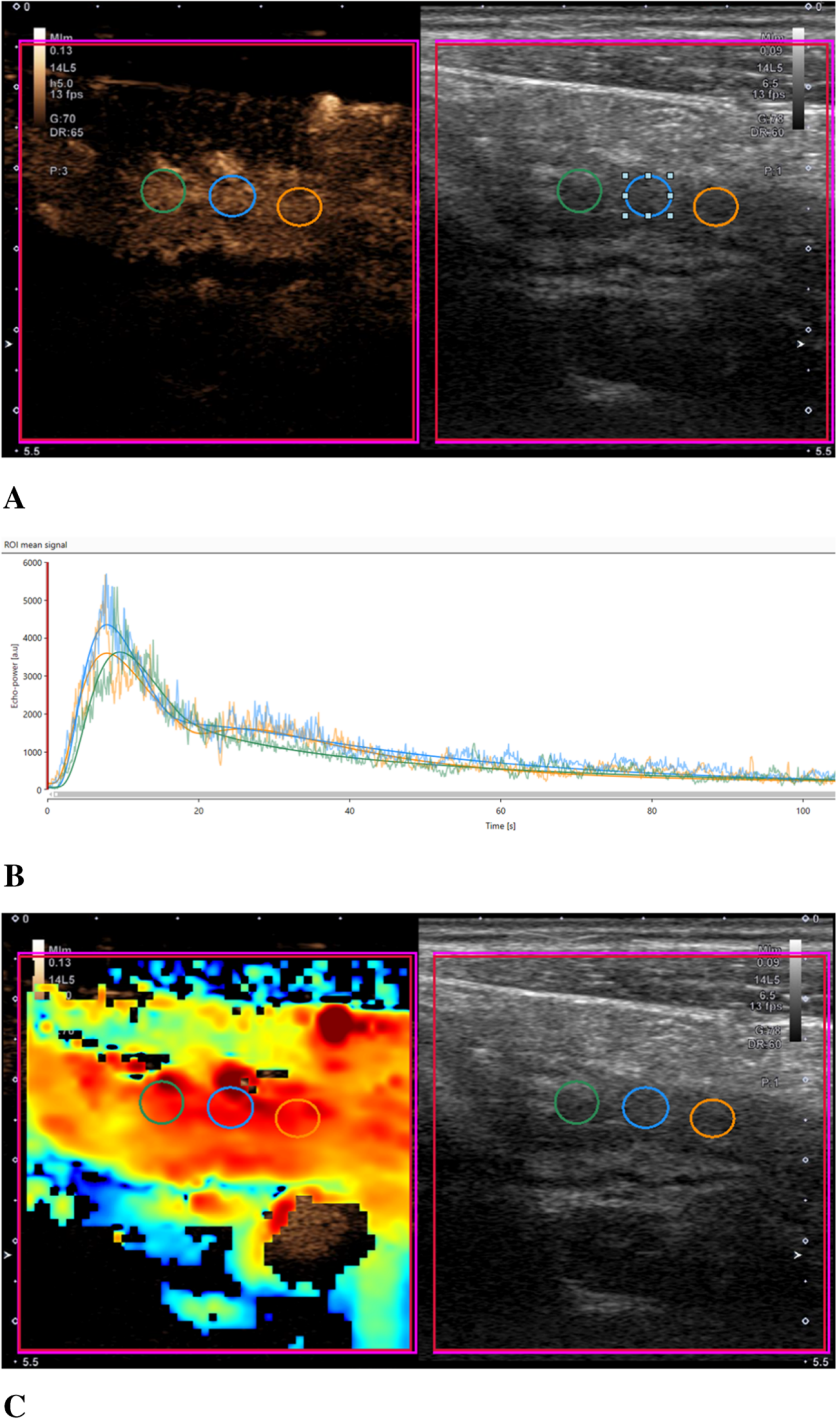
A 16-year-old with ileal Crohn’s disease. **a** Side-by-side contrast-specific and gray-scale contrast-enhanced ultrasound images at the time of bowel wall peak enhancement show mural thickening and relative hyperenhancement when compared to the adjacent mesentery. Three regions of interest were placed in the anterior bowel wall in the area of greatest gray-scale abnormality. **b** Time vs. signal intensity curves from the three regions of interest show bowel wall wash-in and wash-out of microbubble contrast agent. **c** Contrast-enhanced ultrasound peak enhancement parametric map shows relative enhancement of the bowel wall compared to adjacent structures

Dynamic CEUS images were analyzed by a single operator using VueBox® software (Bracco Group) [19] to quantify tissue perfusion (Fig. 1). This operator was blinded to clinical MRI exams and other pertinent clinical and research data. For each participant, three non-overlapping circular regions of interest (ROIs) were placed in the anterior bowel wall, specifically in areas with the most substantial gray-scale abnormality (e.g., area of greatest bowel wall thickening) (Figs. 2 and 3). The ROIs extended from the inner (mucosal) to outer (serosal) surfaces of the bowel wall and varied in size based on the degree of bowel wall thickening. ROI cross-sectional areas were documented. Nine quantitative CEUS metrics were extracted from time versus signal intensity curves for each ROI [19]. The median value for each metric across the three ROIs was calculated for each participant and used in subsequent statistical analyses. The recorded metrics included:

1. Rise time (s), time from the instant at which the maximum slope tangent intersects the *x*-axis to maximum signal intensity within an ROI.
2. Time to peak (s), time from zero signal intensity to maximum signal intensity within an ROI.
3. Fall time (s), time from maximum signal intensity within an ROI to the instant at which the minimum slope tangent intersects the *x*-axis.
4. Mean transit time (local) (s), average time that microbubbles spend within the capillary circulation within an ROI.
5. Average signal intensity (a.u.), average signal intensity within an ROI over the recorded 120-s cine clip.
6. Peak enhancement (a.u.), maximum value of the signal intensity within an ROI.
7. Wash-in rate (a.u.), maximum slope of contrast material wash-in within an ROI.
8. Wash-in area under the curve (WiAUC) (a.u.), area under the time vs. signal intensity curve extending from the instant at which the maximum slope tangent intersects the *x*-axis to the time of peak enhancement.
9. Combined wash-in and wash-out area under the curve (WiWoAUC) (a.u.), total area under the time vs. signal intensity curve.

### Assessment of MRI disease severity

Correlative MRI examinations were separately reviewed by three pediatric radiologists to document features of disease activity. Radiologists were blinded to research CEUS imaging. Multiple MRI findings related to the presence of terminal ileum active inflammation were documented, including maximum bowel wall thickness in millimeters, presence of radiologic ulcers, presence of intramural edema, and presence of perienteric inflammation (fat stranding). Single round ROIs were placed in the bowel wall at the area of greatest signal intensity on axial single-shot fast spin-echo fat-saturated imaging, as well as in the right psoas or iliacus muscle on the same image to measure their respective signal intensities. Bowel wall signal intensity was then normalized to muscle signal intensity to create a “T2 ratio,” which is presumably related to intramural active inflammation. Additional single round ROIs were placed in the area of greatest enhancement on coronal contrast-enhanced (portal venous phase) fat-saturated T1-weighted imaging, and in the right hip or flank subcutaneous fat on the same image to measure their signal intensities. This bowel wall signal intensity was normalized to fat signal intensity to create an “enhancement ratio,” also presumably linked to active intestinal inflammation. Finally, reviewers evaluated the degree of overall active inflammation in the terminal ileum using a visual analog scale (VAS) ranging from 0 mm to 100 mm. On this scale, 0 mm indicated normal, non-inflamed bowel, while 100 mm represented the most severe bowel wall inflammation conceivable.

Continuous variables, including maximum bowel wall thickness, T2 ratio, enhancement ratio, and VAS, were averaged across the three radiologists, and these means were used for statistical analyses. The presence of bowel wall thickening greater than 3 mm, ulcers, intramural edema, and perienteric inflammation was used to calculate the simplified magnetic resonance index of activity (sMaRIA, ranging from 0–5) [20]. sMaRIA scores were also averaged across the three radiologists, with the means utilized for statistical analysis.

### Statistical analysis

Continuous variables were summarized as means, standard deviations, and ranges, while categorical variables were presented as counts and percentages. Pearson’s correlation coefficients (*r*) were used to assess associations between various CEUS quantitative metrics, with a coefficient greater than 0.8 indicating metric collinearity. These coefficients were also used to evaluate the relationships between CEUS quantitative metrics and MRI features of disease activity as well as clinical inflammatory markers. Strengths of correlations were interpreted as follows: 0 to <0.2 very weak; 0.2 to <0.4 weak; 0.4 to <0.6 moderate; 0.6 to <0.8 strong; and 0.8–1 very strong. Intraclass correlation coefficients (ICC) were used to assess inter-reader agreement for semi-quantitative/quantitative MRI features of disease activity.

A *P*-value of less than 0.05 was deemed significant for inference testing, and 95% confidence intervals were calculated, as appropriate. Due to the exploratory nature of this investigation, adjustments for multiple comparisons and formal sample size calculation were not performed. Statistical analyses were performed using GraphPad Prism version 10.0.0 for Windows (GraphPad Software).

## Results

### Study sample

Twenty-five participants were included in our study. The mean age was 16.5 years, and 13 (52%) participants were male. The mean CRP was 1.1 mg/l, while the mean HBI score was 2.6. The mean time between diagnosis with CD and research CEUS imaging was 1.8±3.1 years (range, 1 month to 11.3 years). All participants were receiving medical therapy for their CD, including budesonide (*n*=8), infliximab (*n*=7), adalimumab (*n*=7), prednisone (*n*=1), ustekinumab (*n*=1), and vedolizumab (*n*=1). The study sample is further described in Table 1.

**Table 1:** Description of study sample (*n*=25). Means, standard deviations, and ranges are presented except for biologic sex which is reported using counts and percentages

	Mean	SD	Range
Age (years)	16.5	2.5	13–20
Sex	Female	12 (48%)	
	Male	13 (52%)	
BMI (kg/m^2^)	21.4	3.8	16.4–29.5
CRP (mg/l)	1.1	2.2	0.29–11.2
ESR (mm/h)	14.0	17.0	1–80
Harvey-Bradshaw Index	2.6	1.6	0–6

*BMI*, body mass index; *CRP*, C-reactive protein; *ESR*, erythrocyte sedimentation rate

The mean dose of contrast material injected was 1.8±0.3 ml (range, 1.3–2.4 ml). Research CEUS exams were performed within 41.7±44.0 days (range, 12–106 days) of the clinical MRE exam, on average.

### Summary of MRI disease severity

Terminal ileal mean maximum bowel wall thickness at MRI was 7.5 mm±1.8 mm. The mean sMaRIA score was 3.4±0.8, consistent with severely active disease by MRI on average. The mean VAS was 45.7±19.0. Study sample MRI disease severity features are further described in Table 2.

**Table 2:** Summary of study sample MRI disease activity features

	Mean	SD	Range	ICC
Maximum bowel wall thickness (mm)	7.5	1.8	4.2–11.2	0.76 (0.54–0.88)
sMaRIA (0–5)	3.4	0.8	2–5	0.42 (−0.07-0.72)
T2w SI ratio	3.8	1.4	2.1–8.7	0.62 (0.27–0.82)
Enhancement ratio	11.5	5.0	3.9–26.3	0.57 (0.21–0.79)
Visual analog scale (0–100)	45.7	19.0	21.7–85.0	0.68 (0.13–0.87)

*ICC*, intraclass correlation coefficient; *sMaRIA*, simplified magnetic resonance index of activity; *SI*, signal intensity

### Summary of CEUS quantitative measurements

The terminal ileum was visible and could be assessed in 25/25 (100%) participants. CEUS quantitative measurements were highly variable between participants. The mean rise time was 7.0±2.7 s, while the mean transit time was 54.8±44.1 s. The peak enhancement was 3,282±3,754 a.u., while the mean wash-in AUC was 13,944±14,361 a.u. Study sample CEUS quantitative measurements are further summarized in Table 3.

**Table 3:** Summary of study sample contrast-enhanced ultrasound quantitative measurements

	Mean	SD	Range
Rise time (s)	7.0	2.7	2.3–16.0
Time to peak (s)	10.6	3.7	4.1–20.8
Fall time (s)	15.4	7.5	4.4–37.0
mTT (s)	54.8	44.1	16.9–212.8
Average SI (a.u.)	1,008	1,207	32-4,561
Peak enhancement (a.u.)	3,282	3,754	126-13,456
Wash-in rate (a.u.)	768	927	21-3,013
WiAUC (a.u.)	13,944	14,361	413-46,974
WiWoAUC (a.u.)	45,075	46,758	1,491-170,601

*a.u.*, arbitrary units; *mTT*, mean transit time (local); *SI*, signal intensity; *WiAUC*, wash-in area under the curve; *WiWoAUC*, combined wash-in and wash-out areas under the curve

### Correlations between CEUS quantitative measurements

Twelve of 36 (33%) CEUS quantitative metric bivariate comparisons were highly associated with one another (i.e., collinear) with *r*>0.8 (*P*<0.0001). Correlations between these metrics are presented in Table 4 and Fig. 3.

**Table 4:** Correlations between contrast-enhanced ultrasound quantitative measurements. Results are presented as Pearson’s correlation coefficients (*r*) with 95% confidence intervals in parentheses and *P*-values in brackets (*n*=25). Bold=statistically significant

	Rise time (s)	Time to peak (s)	Fall time (s)	mTT (s)	Average SI (a.u.)	Peak enhancement (a.u.)	Wash-in rate (a.u.)	WiAUC (a.u.)	WiWoAUC (a.u.)
Rise time (s)	1.00								
Time to peak (s)	**0.89** **(0.76–0.95)** **[<0.0001]**	1.00							
Fall time (s)	**0.90** **(0.78–0.95)** **[<0.0001]**	**0.63** **(0.32–0.82)** **[0.001]**	1.00						
mTT (s)	**0.57** **(0.23–0.79)** **[0.003]**	**0.45** **(0.06–0.72)** **[0.03]**	**0.48** **(0.11–0.74)** **[0.01]**	1.00					
Average SI (a.u.)	0.08 (−0.33-0.46) [0.70]	0.15 (−0.26-0.52) [0.47]	−0.04 (−0.43-0.36) [0.84]	0.34 (−0.06-0.65) [0.09]	1.00				
Peak enhancement (a.u.)	−0.003 (−0.40-0.39) [0.99]	0.09 (−0.32-0.47) [0.67]	−0.13 (−0.50-0.28) [0.53]	0.25 (−0.16-0.59) [0.22]	**0.95** **(0.90–0.98)** **[<0.0001]**	1.00			
Wash-in rate (a.u.)	−0.16 (−0.52-0.25) [0.45]	−0.05 (−0.44-0.35) [0.80]	−0.26 (−0.60-0.15) [0.20]	0.20 (−0.22-0.55) [0.35]	**0.91** **(0.79–0.96)** **[<0.0001]**	**0.98** **(0.94–0.99)** **[<0.0001]**	1.00		
WiAUC (a.u.)	0.31 (−0.09-0.63) [0.13]	0.39 (−0.01-0.68) [0.06]	0.13 (−0.28-0.50) [0.53]	**0.45** **(0.07–0.72)** **[0.02]**	**0.89** **(0.77–0.95)** **[<0.0001]**	**0.92** **(0.82–0.96)** **[<0.0001]**	**0.84** **(0.67–0.93)** **[<0.0001]**	1.00	
WiWoAUC (a.u.)	0.34 (−0.06-0.65) [0.10]	0.38 (−0.02-0.67) [0.06]	0.19 (−0.23-0.54) [0.37]	**0.43** **(0.05–0.71)** **[0.03]**	**0.89** **(0.75–0.95)** **[<0.0001]**	**0.91** **(0.81–0.96)** **[<0.0001]**	**0.82** **(0.63–0.92)** **[<0.0001]**	**0.98** **(0.95–0.99)** **[<0.0001]**	1.00

*a.u.*, arbitrary units; *mTT*, mean transit time; *SI*, signal intensity; *WiAUC*, wash-in area under the curve; *WiWoAUC*, combined wash-in and wash-out areas under the curve

### Correlations between CEUS quantitative measurements, MRI disease activity, and clinical inflammatory markers

**Fig. 4:**
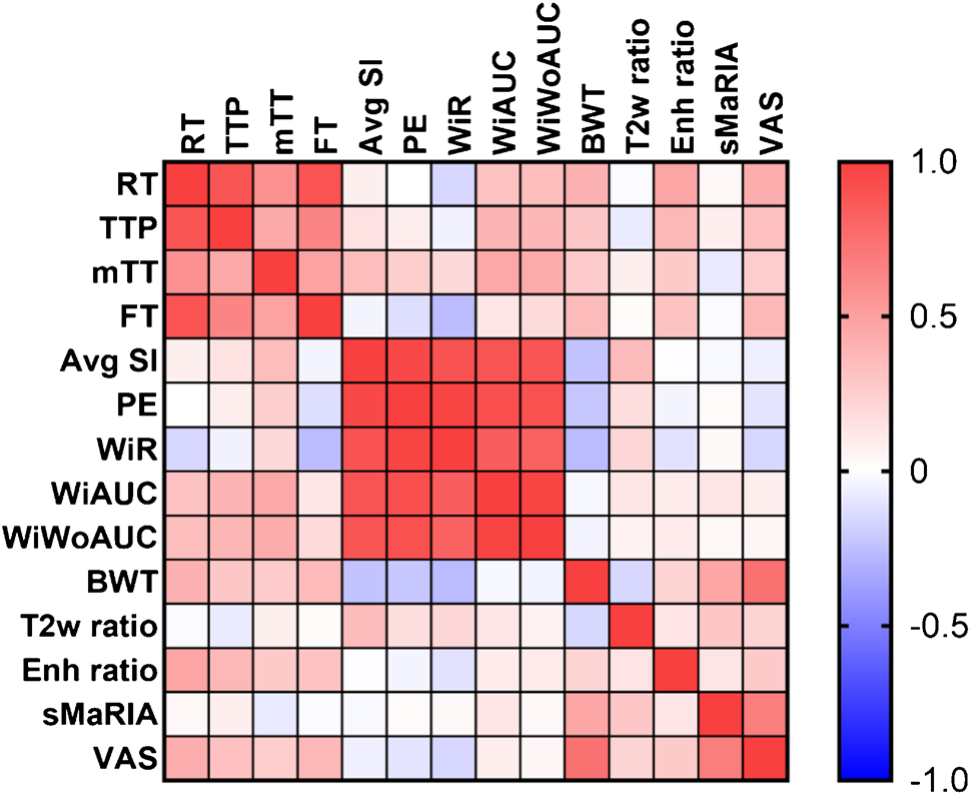
Heatmap showing correlations between contrast-enhanced ultrasound quantitative metrics and MRI features of disease activity. Exact correlation coefficients are presented in Tables 4 and 5. RT, rise time; TTP, time to peak; mTT, mean transit time (local); FT, fall time; Avg SI, average signal intensity; PE, peak enhancement; WiR, wash-in rate; WiAUC, wash-in area under the curve; WiWoAUC, combined wash-in and wash-out area under the curve; BWT, maximum bowel wall thickness; T2w ratio, bowel wall T2-weitghted signal intensity normalized to muscle; Enh ratio, bowel wall enhancement normalized to fat; sMaRIA, simplified magnetic resonance index of activity; VAS, visual analog scale

There were significant positive correlations between CEUS rise time and MRI maximum bowel wall thickness (*r*=0.40; *P*=0.046), visual analog scale assessment of overall inflammation (*r*=0.43; *P*=0.032), and postcontrast enhancement ratio (*r*=0.47; *P*=0.018). There were no other significant correlations between CEUS quantitative metrics and MRI features of disease activity (Table 5 and Fig. 4).

**Table 5:** Correlations between contrast-enhanced ultrasound quantitative measurements and MRI disease activity features. Results are presented as Pearson’s correlation coefficients (*r*) with 95% confidence intervals in parentheses and *P*-values in brackets (*n*=25). Bold=statistically significant

	Maximum bowel wall thickness (mm)	sMaRIA (0–5)	T2w SI ratio	Enhancement ratio	Visual analog scale (0–100)
Rise time (s)	**0.40** **(0.01–0.69)** **[0.046]**	0.04 (−0.36-0.43) [0.86]	−0.02 (−0.41-0.38) [0.92]	**0.47** **(0.09–0.73)** **[0.02]**	**0.43** **(0.04–0.70)** **[0.03]**
Time to peak (s)	0.29 (−0.12-0.61) [0.16]	0.09 (−0.32-0.47) [0.67]	−0.08 (−0.46-0.33) [0.71]	0.38 (−0.02-0.67) [0.06]	0.33 (−0.07-0.64) [0.11]
Fall time (s)	0.36 (−0.04-0.66) [0.08]	−0.02 (−0.41-0.38) [0.92]	0.02 (−0.38-0.41) [0.93]	0.31 (−0.10-0.63) [0.13]	0.38 (−0.02-0.67) [0.06]
mTT (s)	0.27 (−0.14-0.60) [0.19]	−0.09 (−0.47-0.32) [0.67]	0.08 (−0.33-0.46) [0.70]	0.28 (−0.13-0.61) [0.18]	0.26 (−0.15-0.59) [0.21]
Average SI (a.u.)	−0.24 (−0.58-0.17) [0.26]	−0.03 (−0.42-0.37) [0.88]	0.35 (−0.06-0.65) [0.09]	−0.01 (−0.40-0.39) [0.97]	−0.07 (−0.45-0.34) [0.75]
Peak enhancement (a.u.)	−0.22 (−0.57-0.19) [0.29]	0.03 (−0.37-0.42) [0.91]	0.18 (−0.23-0.53) [0.40]	−0.05 (−0.43-0.36) [0.83]	−0.11 (−0.48-0.30) [0.61]
Wash-in rate (a.u.)	−0.27 (−0.60-0.14) [0.19]	0.03 (−0.37-0.42) [0.89]	0.21 (−0.21-0.56) [0.33]	−0.12 (−0.49-0.29) [0.58]	−0.16 (−0.52-0.25) [0.45]
WiAUC (a.u.)	−0.03 (−0.42-0.37) [0.88]	0.13 (−0.28-0.50) [0.54]	0.13 (−0.28-0.50) [0.54]	0.10 (−0.31-0.47) [0.65]	0.09 (−0.32-0.46) [0.69]
WiWoAUC (a.u.)	−0.05 (−0.44-0.35) [0.82]	0.04 (−0.36-0.43) [0.86]	0.06 (−0.34-0.45) [0.77]	0.10 (−0.31-0.48) [0.63]	0.04 (−0.36-0.43) [0.84]

*a.u.*, arbitrary units; *mTT*, mean transit time; *SI*, signal intensity; *sMaRIA*, simplified magnetic resonance index of activity; *WiAUC*, wash-in area under the curve; *WiWoAUC*, combined wash-in and wash-out areas under the curve

There were no significant correlations between any CEUS metric and either C-reactive protein, erythrocyte sedimentation rate, or HBI (all *P*-values >0.1). There were positive associations between C-reactive protein and erythrocyte sedimentation rate (*r*=0.85; *P*<0.0001) and between C-reactive protein and HBI (*r*=0.45; *P*=0.02), while there was no significant correlation between erythrocyte sedimentation rate and HBI (*r*=0.25; *P*=0.24).

## Discussion

The findings of this study contribute important insights into the potential use of CEUS in assessing CD in pediatric and young adult populations. Our study demonstrates that several CEUS quantitative metrics exhibit significant (multi)collinearity, with 33% (12 of 36) of the bivariate comparisons yielding a very strong correlation. This suggests that certain metrics may provide redundant information about intestinal perfusion and disease activity. Identifying these interrelationships is crucial, as it may allow clinicians to streamline the number of metrics assessed during CEUS evaluations, simplifying interpretation and potentially reducing the time required for image analysis. A review of the heatmap in Fig. 3 suggests that two groups of metrics are highly correlated, with metrics related to time (e.g., time to peak, rise time, fall time) and degree of bowel wall enhancement (e.g., average signal intensity, peak enhancement, areas under the curve) showing a high degree of relatedness. There was also notable variability observed in CEUS measurements among participants that could be due to technical factors versus the heterogeneity of the disease process itself. This variability deserves further study.

Significant positive correlations were observed between CEUS rise time and key MRI features, including maximum bowel wall thickness, visual analog scale (VAS) assessment of overall inflammation, and postcontrast enhancement ratio. These correlations support the supposition that CEUS might serve as an adjunct to MRI in assessing the severity of intestinal inflammation in CD, potentially even in the clinic. The rise time in CEUS, which reflects the time taken for contrast agent to peak within an ROI, appears to moderately correlate with the degree of disease activity as indicated by MRI. This finding highlights the potential of CEUS to provide complementary information regarding the dynamics of bowel perfusion in relation to structural changes observed on gray-scale ultrasound.

However, other CEUS quantitative metrics did not show significant correlations with MRI features of disease activity. This lack of correlation may suggest that some quantitative metrics may not capture aspects of inflammation as effectively as others, or that MRI remains superior in depicting certain characteristics of the disease. With regard to multiphase contrast-enhanced MRE, it is possible that we are not measuring peak enhancement of the bowel wall, as it may occur between the obtained imaging phases or is underestimated due to the length of image volume acquisition. Further studies are needed to delineate the specific contributions of each CEUS metric, both alone and in conjunction with other CEUS metrics as well as with gray-scale and Doppler findings that correlate with MRI disease activity [21], and to explore their clinical utility and associations with meaningful outcomes.

There is a general lack of published studies systematically evaluating CEUS metrics in children and young adults with CD. Mudambi et al. performed CEUS of the bowel in 20 children, including 16 with known CD. In their study, subjective intestinal hyperenhancement was highly sensitive for inflammation, although no quantitative assessments were obtained [11]. Ponorac et al. performed CEUS on 40 intestinal segments from 24 children with CD [12]. In their study, CEUS peak enhancement had a moderate sensitivity of 72.2% and diagnostic accuracy of 87.5% in predicting moderate or severe inflammation at histopathology compared to 55.6% and 72.5%, respectively, for combined gray-scale and color Doppler ultrasound evaluation. In 127 adult patients with CD, Medellin-Kowalewski et al. showed 76% concordance between CEUS and color Doppler ultrasound, with CEUS peak enhancement positively correlating with increasing bowel wall thickness [15]. In 105 adult patients with CD and ileal involvement, Lu et al. showed no correlation between CEUS peak enhancement and histologic active inflammation; a moderate positive correlation was observed between peak enhancement and chronic inflammation (*r*=0.6; *P*=0.03) [16].

The integration of CEUS into routine clinical practice for monitoring CD offers potential advantages, particularly in terms of patient comfort and healthcare costs. CEUS does not require ionizing radiation or sedation, making it particularly suitable for pediatric patients who may be more vulnerable to the potential long-term effects of these exposures. Additionally, these characteristics as well as the ease of performing CEUS could allow for more frequent, objective assessments, which is invaluable in managing chronic conditions like CD, where disease activity can change over time and longitudinal monitoring is likely associated with outcomes [22]. However, there also remains a general paucity of data in children comparing CEUS to more conventional gray-scale and Doppler ultrasound assessments in the clinic, including in the longitudinal setting. Such research should be prioritized to define the exact benefits of CEUS, as CEUS does require placement of an intravenous catheter and comes with a small but real risk of contrast-associated adverse events. Furthermore, the number of bowel segments that can be quantitatively assessed by CEUS (typically one or two) is limited when compared to conventional ultrasound based on the number of contrast material injections that can be administered during a single setting based on the currently approved prescribing information.

This study has several limitations. The exploratory nature of the research, coupled with a relatively small sample size, potentially limits the generalizability of our findings. Larger studies in both children and adults are needed to confirm our observations. Furthermore, our cross-sectional study design restricts our ability to draw conclusions about the temporal relationships between CEUS metrics and disease activity, including the diagnostic performance of CEUS for longitudinal monitoring. CEUS exams were analyzed by a single operator. The reliability of the various CEUS quantitative metrics remains unknown, as inter-operator agreement was not assessed, and will be the subject of future research by our group. While our study evaluated associations between CEUS quantitative metrics and MRI, we did not correlate CEUS with histology or meaningful clinical outcomes. Furthermore, there were about 42 days, on average, between the two exams, with all participants receiving medical treatment during this time period. Finally, all CEUS exams were performed using a single ultrasound system, a single weight-based contrast material dosing algorithm, and were processed using a single analysis software platform. Additional research is needed to understand the impact of using different ultrasound systems, higher and lower doses of contrast material, and different analysis software platforms on measurement reproducibility.

In conclusion, our study highlights the potential of CEUS as a tool for evaluating CD in children and young adults. Multiple CEUS quantitative metrics related to intestinal perfusion are highly inter-related, and it is possible that only a small number of metrics are needed to evaluate the bowel of patients with CD, thereby simplifying interpretation and potentially reducing the time required for image analysis. The demonstrated correlations between CEUS rise time and MRI features of disease activity also warrant further investigation. Integrating CEUS with traditional modalities like CTE, MRE, and endoscopy could enhance the monitoring and care of patients with CD, especially due to its nonionizing and relatively noninvasive nature and potential benefits in terms of cost and accessibility, although additional research is needed.

## Data Availability

Data is available upon reasonable request.
